# Insomnia and Suffering

**Published:** 1898-11

**Authors:** 


### Insomnia and Suffering.

The recent lecture by Dr. E. Symes Thompson, Gresham Professor of Medicine, on "Sleeplessness and Suffering," attracted a large audience to Gresham College, the interest being intensified, perhaps, because the lecture in question was the fourth and concluding one of the series. Referring to his explanations in a previous lecture on the nearly bloodless condition of the brain during sleep, Dr. Thompson pointed out that the consequence was that in sleep the circulation of blood in the brain was very much diminished, and the activity of the brain becoming less, sleep occurred. It followed also that any conditions tending to promote an active circulation of blood in the brain were more or less unfavorable to sleep. This, Dr. Thompson pointed out, happened when any portion of the brain, especially if it was an important part of it, were affected in that way, as would result from long and continuous work, and particularly if that work were associated with any kind of worry. Wakefulness is, in fact, due to a violation of the laws in accordance with which the healthy circulation of blood through the brain proceeds. So far as sleeplessness might be the result of retention in the system of the waste products of energy, the lecturer observed that to some extent relief might be obtained by means of draughts of cold water. With regard to the allowance of sleep we ought to give ourselves, it was pointed out that we should rise when we woke with a healthy sense of refreshment and relaxation. When we get into a disordered state of sleeping, it is time for us to face the facts, and endeavor to remove the causes of the irregularity. Not the least interesting portion of a singularly striking lecture was the exhibition of an anatomical diagram, showing the complex nervous connections of the brain, the heart and the stomach, and the accompanying remarks on their practical value as medical guides. Equally to the point was the lecturer's caution against the use of strong drugs, though they might appear to be very useful and quickly effective. Hydrocyanic acid, he remarked, acted with lightning-like rapidity, sometimes killing in two minutes, but there were other valuable deterrents, which were unattended with serious danger. Nitrous oxide gas will produce insensibility to pain, but that insensibility might afterwards be maintained by means of ether. In regard to the best posture to assume during slumber, the lecturer reiterated his remarks in counselling his hearers to sleep on their right sides, as it would favor the peristaltic motion.—*Indian Lancet.*

